# MicroRNA-142-3p promotes renal cell carcinoma progression by targeting RhoBTB3 to regulate HIF-1 signaling and GGT/GSH pathways

**DOI:** 10.1038/s41598-022-21447-2

**Published:** 2023-04-12

**Authors:** Yijing Zhang, Sha Ma, Jun Zhang, Lu Lou, Wanqi Liu, Chao Gao, Long Miao, Fanghao Sun, Wei Chen, Xiliang Cao, Jin Wei

**Affiliations:** 1 Department of Urology, China University of Mining and Technology, Xuzhou No.1 People’s Hospital, Xuzhou, China; 2 Department of Hematopathology, The Affiliated Hospital of Xuzhou Medical University, Xuzhou, China; 3 Department of Pulmonary and Critical Care Medicine, Yantaishan Hospital, Yantai, China

**Keywords:** Cancer, Computational biology and bioinformatics, Molecular biology, Biomarkers, Oncology, Urology

## Abstract

MicroRNAs play a critical regulatory role in different cancers, but their functions in renal cell carcinoma (RCC) have not been elucidated. Reportedly, miR-142-3p is involved in the tumorigenesis and the development of RCC in vitro and is clinically correlated with the poor prognosis of RCC patients. However, the molecular target of miR-142-3p and the underlying mechanism are unclear. In this study, we found that miR-142-3p was upregulated in RCC tumor tissues and downregulated in exosomes compared to normal tissues. The expression of miR-142-3p was inversely associated with the survival of patients with kidney renal clear cell carcinoma (KIRC). RhoBTB3 was reduced in RCC, and miR-142-3p plays an inverse function with RhoBTB3 in KIRC. The direct interaction between RhoBTB3 and miR-142-3p was demonstrated by a dual luciferase reporter assay. miR-142-3p promoted metastasis in the xenograft model, and the suppression of miR-142-3p upregulated RhoBTB3 protein expression and inhibited the mRNAs and proteins of HIF1A, VEGFA, and GGT1. Also, the miR-142-3p overexpression upregulated the mRNA of *HIF1A*, *VEGFA*, and *GGT1*. In conclusion, miR-142-3p functions as an oncogene in RCC, especially in KIRC, by targeting RhoBTB3 to regulate HIF-1 signaling and GGT/GSH pathways, which needs further exploration.

## Introduction

RCC, renal cell carcinoma, is a high-risk kidney malignant neoplasm in the proximal convoluted tubules’ lining and has a high mortality rate. It is responsible for nearly 90% of all kidney cancers^1^. RCC is the second most common genitourinary malignant tumor in china, and it’s incidence has been rapidly rising in recent years^2^. Traditional chemotherapy is frequently the first line of treatment for individuals with RCC, but due to tumor resistance to anticarcinogens, results are often moderate or limited. Thus, understanding the pathophysiology of the disease’s advanced aggressive stage is critical for the development of new RCC therapy options^3^.

Hypoxia is a pathological characteristic of solid tumors that gets a lot of attention. HIF-1 (the hypoxia-inducible factor 1) signaling pathway is an oxygen dependent mechanism that response to hypoxia^4^. HIF signaling is activated in response to hypoxia, and HIF target genes, such as those involved in cell angiogenesis, invasion, metastasis, survival, proliferation, cancer stemness, and metabolic reprogramming, are elevated in expression^5,6^. In hypoxia or malfunctioning pVHL (as in KIRC, due to mutation), HIF-1α accumulates and transfers into the nucleus, resulting in HIF-α binding to DNA and subsequent stimulation of the expression of target gene, including *VEGF*^7^.

KIRC is defined histologically by using a “clear-cell” phenotype prompted by lipid and glycogen accumulation, implying that altered fatty acid and glucose metabolism is essential in the improvement of this cancer^8^. Surprisingly, high GSH levels are a major contributor to chemoresistance, which is a major treatment restriction in KIRC^9^. GSH is plentiful in cells, protects against oxidative stress, and keeps the redox balance^10,11^. GGT1 is also known as gamma-glutamyl transpeptidase (GGT) and is involved in maintaining GSH homeostasis and oxidative stress defense^12^.

In patients with genitourinary cancer, serum GGT could be a predictive biomarker^13,14^. Furthermore, in RCC patients, elevated serum GGT levels are linked to a worse prognosis and survival^15–17^, suggesting that modulating the GSH-based antioxidant system, especially thru GGT1 activity, ought to be a promising therapeutic method for overcoming chemoresistance and slowing the development of KIRC^18^. Cisplatin, in contrast to GSH, types adducts with cysteinyl glycine, a byproduct of GGT1 activity, faster^19^. This finding recommended that improved GGT1 activity is to blame for chemotherapeutic resistance, as properly as elevated metastatic capacity and RCC proliferation.

MicroRNAs (miRNAs) suppress the expression of their target mRNAs to regulate cellular signaling^20^, and the resulting interaction network between them and their downstream effectors has a considerable impact on the cellular function regulation^21^. miRNAs play a role in the pathogenesis of RCC as well as the response of patients to various therapeutic modalities. Furthermore, because miRNAs are detectable in patients’ blood and urine, they facilitate the development of noninvasive methods for RCC diagnosis. There are currently no miRNAs that could be widely used as biomarkers or treatment targets in clinical settings.

Dysregulation of miR-142-3p has been associated with a multitude of malignancies^22–30^. In RCC patients following the surgery, elevated expression of miR-142-3p predicts poor prognosis^31^, and it’s linked to more cellular proliferation, less apoptosis and more migration in RCC^32^. However, the molecular substrate of miR-142-3p, as well as the underlying mechanism behind it, are unknown.

The current findings imply that miR-142-3p operates as an oncogene by targeting RhoBTB3 via the HIF-1 signaling and GGT/GSH pathway and that it could be a potential target for RCC treatment, particularly for KIRC.

## Materials and methods

### Cell culture and transfection

The human RCC cell line 786-O was purchased from the Chinese Academy of Sciences Cell Bank of Type Culture Collection (Shanghai, China) and cultured as a monolayer in Dulbecco’s modified Eagle’s medium (DMEM, Gibco, Langley, OK, USA) containing 10% fetal bovine serum (FBS, Gibco), and 1% penicillin–streptomycin (GE Healthcare Life Sciences, HyClone Laboratories, Utah, USA) at 37 °C in a humid atmosphere containing 5% CO_2_ (Thermo Fisher Scientific Inc., MA, USA). miRNA-142-3p inhibitors, miR-142-3p-mimics, and miR-control RNA were designed and synthesized by GeneChem Company (Shanghai, China). The miR-control was the empty lentiviral system with no insertion. 786-O cells were transfected with 50 nM of miRNA-142-3p inhibitors, miR-142-3p-mimics, and miR-control using Lipofectamine 2000 according to the manufacturer’s instructions (Invitrogen, USA). After 48 h, the cells were harvested for further analyses.

### Western blot assay

Cells were collected and lysed with RIPA buffer containing phenylmethanesulfonyl-fluoride (Beyotime, China). Equivalent amounts of total protein were separated by 10% SDS-PAGE (Servicebio, China) and transferred to the PVDF membrane (Servicebio). Then, the membranes were blocked in TBST containing 5% nonfat milk and probed with primary antibodies at 4 °C overnight, followed by incubation with secondary antibodies at room temperature for 1 h. Finally, the immunoreactive bands were developed and examined on the gel imager. The primary antibodies were as follows: RhoBTB3 (AF9183), HIF1A (AF1009), GGT1 (DF6610), VEGFA (AF131), andβ-actin (AF7018) from Affinity.

### Quantitative real-time polymerase chain reaction (qRT-PCR)

Total RNA was extracted from cell lines using TRIzol reagent (Invitrogen). Complementary DNA was synthesized using the TaqManH MicroRNA Reverse Transcription Kit (ABI, CA, USA) or PrimeScript RT Reverse transcriptase reagent kit according to the manufacturer’s instructions (Takara). qRT-PCR was performed using the SYBR Premix Ex Taq kit (Takara) and a Step One Plus Real-Time PCR (Applied Biosystems, USA). The *miR-142-3p*, *RhoBTB3*, *HIF1A*, *VEGFA*, and *GGT1* expression was normalized to that of U6 or *GAPDH*, respectively. The 2^−△△Ct^ method was used to determine the relative expression of the target genes. The has-miR-142-3p precursor and has-miR-142-3p inhibitor and their empty vector lentivirus (GV209, GV309,GV280,GV159, respectively) were purchased from GeneChem (Shanghai, China). Lentiviruses were transduced into cells at a multiplicity of infection (MOI) of 10 in the presence of polybrene. MiRNA expression was detected using the miRcute miRNA qPCR Detection Kit (Tiangen Biotech, China) in a 7500 Real-Time PCR system (Applied Biosystems, CA, USA). The specific sequences of primers used in the qPCR process were designed and synthesized by the stem loop reverse transcription, shown as follows:

*miR-142-3p *(RT):5′-CTCAACTGGTGTCGTGGAGTCGGCAATTCAGTTGAGTCCATAAA-3′

*miR-142-3p* (forward):5′-ACACTCCAGCTGGGTGTAGTGTTTCCTACTT-3′,

*miR-142-3p* (reverse): 5′-TGGTGTCGTGGAGTCG-3′;

*RhoBTB3* (forward): 5′-ATGGACGCTGACATGGACTAC-3′,

*RhoBTB3* (reverse): 5′-ATCCCGAGAACGCTCCAAGA-3′;

*HIF1A* (forward): 5′-ATCCATGTGACCATGAGGAAATG-3′,

*HIF1A* (reverse): 5′-TCGGCTAGTTAGGGTACACTTC-3′;

*VEGFA* (forward): 5′-AACTTTCTGCTGTCTTGGGT-3′,

*VEGFA* (reverse): 5′-TCTCGATTGGATGGCAGTA-3′;

*GGT1* (forward): 5′-CTTGTGTGAGGTGTTCTGCC-3′,

*GGT1* (reverse): 5′-CAGGTCCTCAGCTCAGCTGTCACAA-3′;

*U6* (forward): 5′-CGAGCACAGAATCGCTTCA-3′,

*U6* (reverse): 5′-CTCGCTTCGGCAGCACATAT-3′;

*GAPDH* (forward): 5′-GGTGGCAGAGGCCTTTG-3′,

*GAPDH* (reverse): 5′-TGCCCATTTAGCATCTCCTT-3′.

### Database analysis

The review of miR-142-3p, RhoBTB3, and GGT1 expression in RCC are available in the StarBase [ENCORI: The Encyclopedia of RNA Interactomes. (sysu.edu.cn)].The correlations between miR-142-3p and gene expression data of predicted targets are available in TargetScan (TargetscanHuman 8.0: predicted miRNA targets of miR-142-3p.1), DIANA [DIANA tools—Tarbase v8 (uth.gr)], and miRDB (miRDB Search Result). Furthermore, the target genes predicted by all three databases with the target score 70–100 were identified using the venn diagram which are available in the genevenn [GeneVenn (sourceforge.net)].The functions involving the predicted genes are available in GENEMANIA (GeneMANIA). The expression levels of exosome and tissue miR-142-3p are available in BBCancer [BBCancer (renlab.org)].

### Dual-luciferase reporter assay

The miR-142-3p’s mature sequences targeting of human RHOBTB3 are available in the TargetScan (TargetScanHuman 8.0 predicted targeting of Human RHOBTB3).Plasmids containing the wild-type (WT) or mutated (MUT) fragment of RhoBTB3 3′-UTR mRNA, which contains a putative miR-142-3p complementary site, were synthesized by GeneChem Company (Shanghai, China). Plasmid DNA (WT-RhoBTB3 or MUT-RhoBTB3) and miR-142-3p or NC mimics were co-transfected into 786-O cells according to the manufacturer’s instructions. After 24 h of transfection, luciferase activity was determined by the Dual-Luciferase Reporter Assay System (Promega, Madison, WI, USA) according to the manufacturer’s instructions.

### Xenograft model

Female nude mice, 5–6-weeks-old were housed in specific pathogen-free (SPF)-grade facilities with controlled humidity and room temperature. All animal protocols were carried out in accordance with the experimental procedures and requirements issued by Jiangsu Provincial Association for Experimental Animals. The animals were fed in the isolation cages with access to food and water ad libitum. After 7 days of culture in the SPF environment, miR-control, miR-142-3p-mimics, and miR-142-3p-inhibitors transfected 786-O cells were injected in the mice via tail vein. Each mouse was injected with 3 × 10^6^ cells (one died after injection). A total of 12 mice were divided into three experimental groups. Animal experiments were approved by the Institutional Animal Care and Use Committee of China University of Mining and Technology, Xuzhou No.1 People’s Hospital (Protocol number xyyll[2021]019). After 9 weeks, one mouse from each group was randomly selected for micro-computed tomography (micro-CT). The exposure field was selected as the lung, approximately 1 cm in length. The larger the relative CT value, the denser the lung tumor tissue of the mice. On day 2, the mice were sacrificed, and the lung tissues were harvested, fixed in 4% paraformaldehyde, embedded in paraffin, and sectionalized for hematoxylin–eosin (HE) and immunohistochemistry (IHC) staining. The processed paraffin sections were incubated with primary antibodies against GGT1, HIF1A, RhoBTB3, and VEGFA, followed by incubation with the corresponding secondary antibodies. The sections were photographed under a normal microscope to assess the staining intensity. Our study is reported in accordance with ARRIVE guidelines (PloS Bio 8(6), e1000412, 2010) (https://arriveguidelines.org/).

### Statistical analysis

SPSS 19.0 statistical software (SPSS Inc., USA) was used to analyze the data. The experiments were performed in triplicate. Data are presented as the mean ± standard deviation (SD). Student’s t-test (two-tailed) and one-way analysis of variance (ANOVA) were performed to analyze the data, and significance was indicated as *P < 0.05, **P < 0.01, ***P < 0.001, and not significant (NS) as *P* > 0.05.

## Results

### Expression of miR-142-3p in RCC

**Figure 1:**
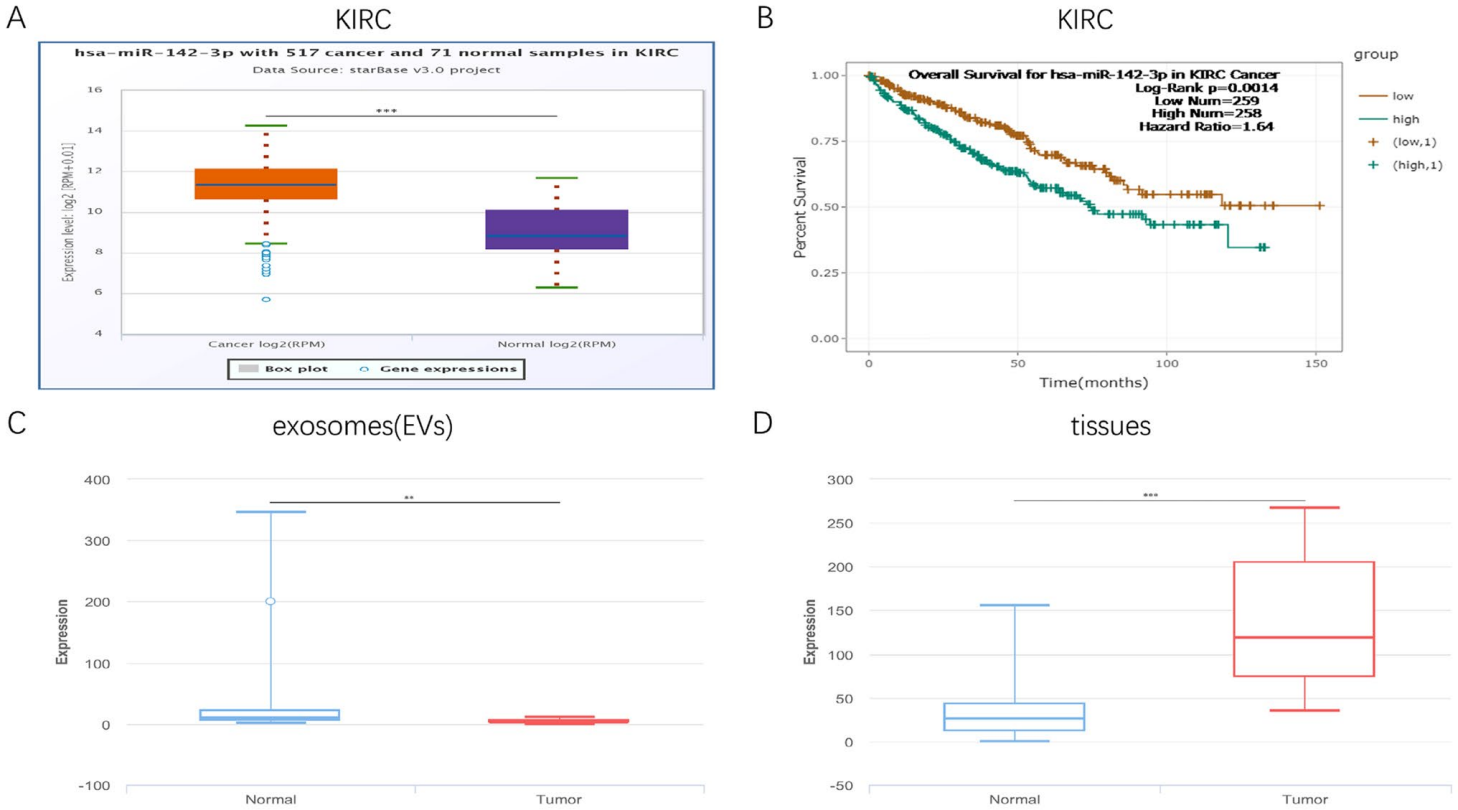
Expression pattern of miR-142-3p in clinical samples. Expression of miR-142-3p in KIRC (**A**, *P* < 0.001) samples compared to adjacent normal tissues. Kaplan–Meier survival curves analysis for patients in TCGA KIRC (**B**, *P* < 0.01) cohort (Orange = low, Blue = high). Expression of miR-142-3p in renal tumor patients in EVs (**C**, *P* < 0.01) and tissues (**D**, *P* < 0.001) compared to normal patients.

First, we determined the expression level of miR-142-3p in all RCC subtypes by using StarBase. As shown in Fig. 1A and Supplementary Fig. 1A, higher miR-142-3p expression was detected in KIRC and KIRP samples compared to the adjacent normal tissues (*P* < 0.001). In Supplementary Fig. 1C, the expression level of miR-142-3p did not differ in KICH between adjacent normal tissues. Kaplan–Meier survival analyses demonstrated that the expression of miR-142-3p was negatively associated with the survival of patients with KIRC (Fig. 1B, *P* < 0.01) but was disassociated with the survival of patients with KIRP and KICH (Supplementary Fig. 1B,D). Then, the expression level of miR-142-3p exosomes and tissues was predicted by using BBCancer. Although a higher level of miR-142-3p was detected in renal tumor tissues (Fig. 1D), the level of miR-142-3p was significantly reduced in renal tumors compared to the normal (Fig. 1C) exosomes or extracellular vesicles (EVs).

### Negative correlation between miR-142-3p and RhoBTB3 in KIRC

**Figure 2:**
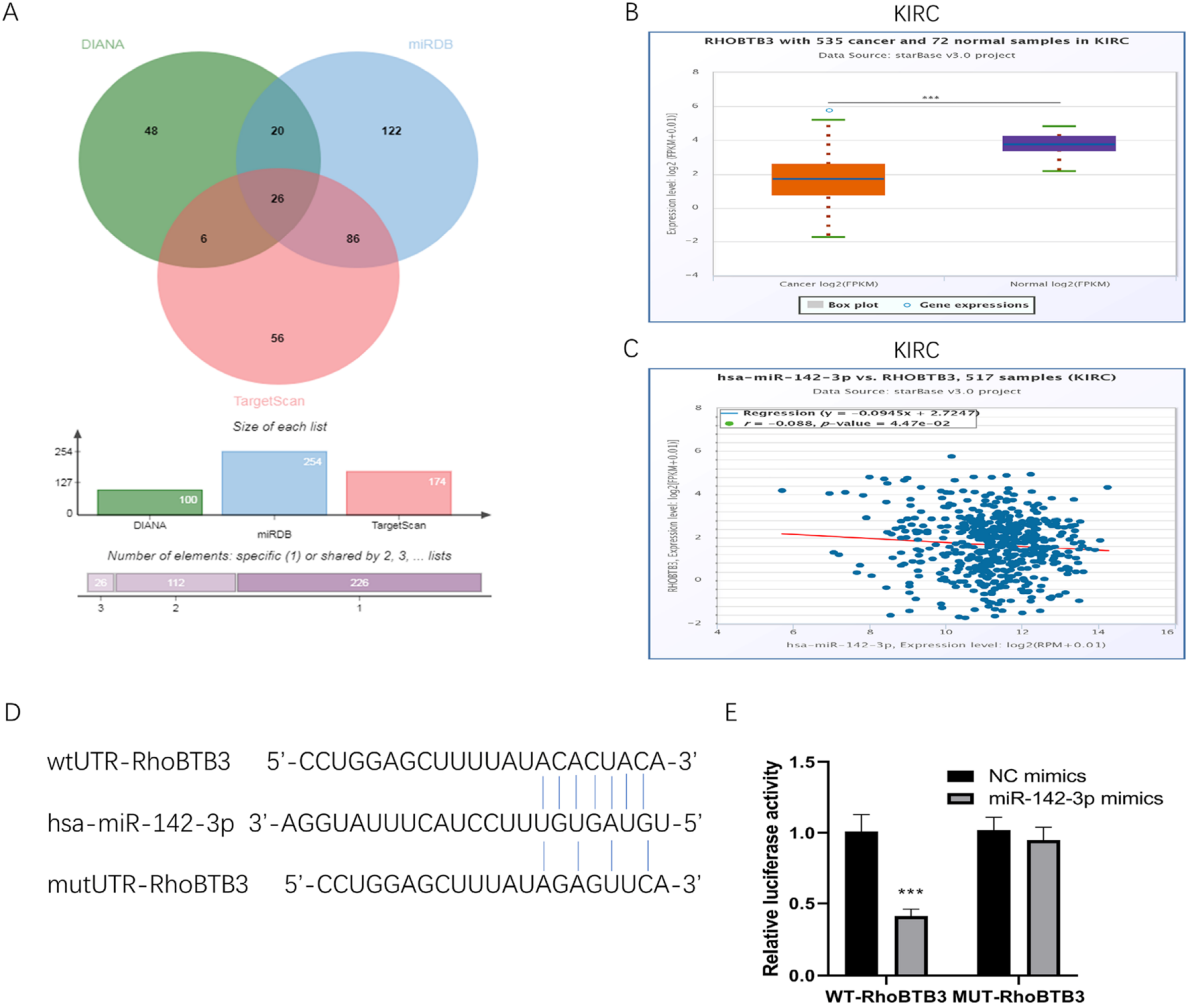
Database analysis of miR-142-3p and RhoBTB3. Venn diagram depicts the mRNA targets of miR-142-3p predicted by three independent algorithms, Target Scan, DIANA, and miRDB (**A**). The expression of RhoBTB3 was reduced significantly in KIRC (**B**, *P* < 0.001). A negative correlation was established between miR-142-3p and RhoBTB3 in KIRC (**C**, *P* < 0.05). TargetScan software indicated that RhoBTB3 is a target gene of miR-142-3p (**D**). A luciferase reporter assay was conducted on 786-O cells to confirm the relative luciferase activity of RhoBTB3 after treatment with miR-142-3p mimics (**E**).

The correlation between miR-142-3p and gene expression data of predicted targets using Target Scan, DIANA, and miRDB was analyzed to identify the potential miR-142-3p target genes. The target genes predicted by all the three databases were identified using the venn diagram powered by the genevenn. As shown in venn diagram (Fig. 2A), 26 genes were predicted as targets in all three databases. The genes and their miRDB target score are as follows: *RHOBTB3* (99), *ASH1L* (98), *CLOCK* (98), *ZCCHC14* (95), *UTRN* (95), *RGL2* (95), *WASL* (94), *C16orf70* (93), *STRN3* (92), *BNC2* (92), *TGFBR1* (89), *SMG1* (89), *TNKS* (88), *CCDC6* (88), *GAB1* (88), *PCGF3* (86), *C90rf72* (86), *ROCK2* (85), *PUM1* (84), *NR2F6* (83), *IRAK1* (83), *IPMK* (82), *RERE* (81), *RARG* (81), *GNAQ* (80), and CNIH4 (75). To further determine whether RhoBTB3 is a prognostic marker for RCC, Kaplan–Meier survival analysis and the log-rank test was performed. The expression of RhoBTB3 was reduced significantly in RCC (KIRC, Fig. 2B; KIRP, Supplementary Fig. 2A; KICH, Supplementary Fig. 2C, all *P* < 0.001). Furthermore, a negative correlation was established between miR-142-3p and RhoBTB3 in KIRC (Fig. 2C, *P* < 0.05), suggesting that miR-142-3p and RhoBTB3 play an inverse function in renal cancer cells. However, in KIRP and KICH, the correlation between the miR-142-3p and RhoBTB3 did not show a significant difference (Supplementary Fig. 2B,D, *P* > 0.05). We first determined the complementary sequence of miR-142-3p in the 3′-UTR of RhoBTB3 mRNA using targetScan software (Fig. 2D). Next, we tested the direct binding interaction between miR-142-3p and RhoBTB3 using a luciferase reporter assay in 786-O cells. The luciferase intensity of WT-RhoBTB3 cells was significantly reduced following transfection with miR-142-3p mimics compared with NC mimics (*P* < 0.001), while MUT-RhoBTB3 abolished this influence (*P* > 0.05) (Fig. 2E). Taken together, these data indicate that RhoBTB3 is a direct target of miR-142-3p.

### miR-142-3p regulates RCC metastasis in vivo

**Figure 3:**
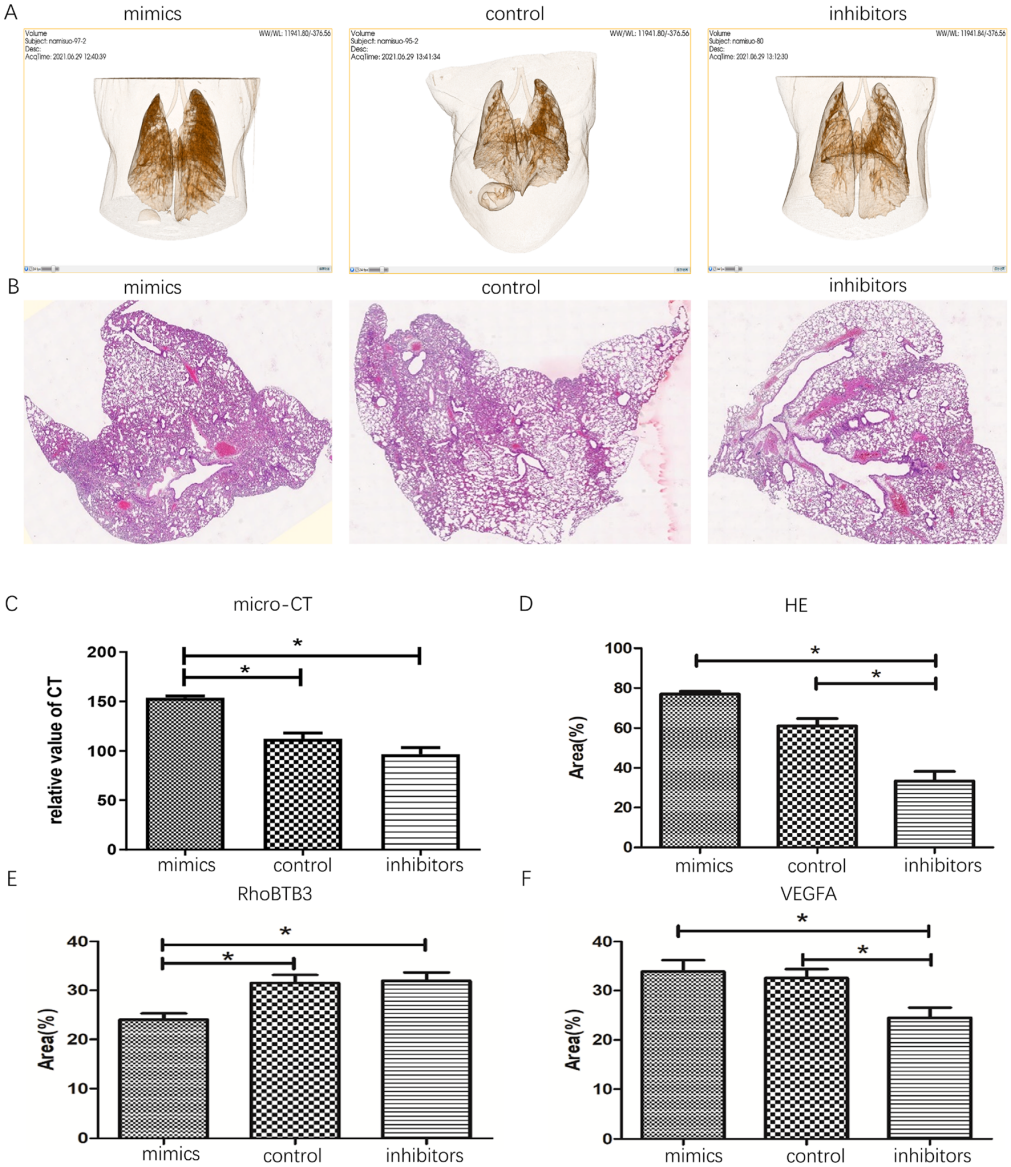
miR-142-3p regulates RCC metastasis in vivo. 786-O cells were transfected with miR-control, miR-142-3p-mimics, and miR-142-3p-inhibitors and implanted into the nude mice. The lung metastasis imaging by micro-CT (**A**) and the relative CT value was analyzed using Image J software (**C**, P < 0.05). The HE stained sections were photographed under the microscope (**B**), and the expression area was also assessed statistically (**D**, P < 0.05). The IHC staining was used to validate the expression of RhoBTB3 and VEGFA in the tissues (**E**,**F**).

We further verified whether miR-142-3p regulates the progression of RCC in vivo. 786-O cells transfected with miR-control, miR-142-3p-mimics, and miR-142-3p-inhibitors were subcutaneously inoculated into female nude mice. Micro-CT results showed that lung metastases of mice in the mimics group are significantly higher than those in control and the inhibitors groups (Fig. 3A,C; *P* < 0.05). Then, mice were sacrificed, and tumor tissues were subjected to HE and IHC staining. The HE staining showed that lung metastases in mice in the mimics and control group were significantly higher than those in the inhibitors group (Fig. 3B,D; *P* < 0.05). IHC showed that the expression of RhoBTB3 and VEGFA was negatively and positively correlated with miR-142-3p in tissues, respectively (Fig. 3E,F; *P* < 0.05).

### miR-142-3p regulates RCC migration and invasion via hypoxia and GSH pathways

**Figure 4:**
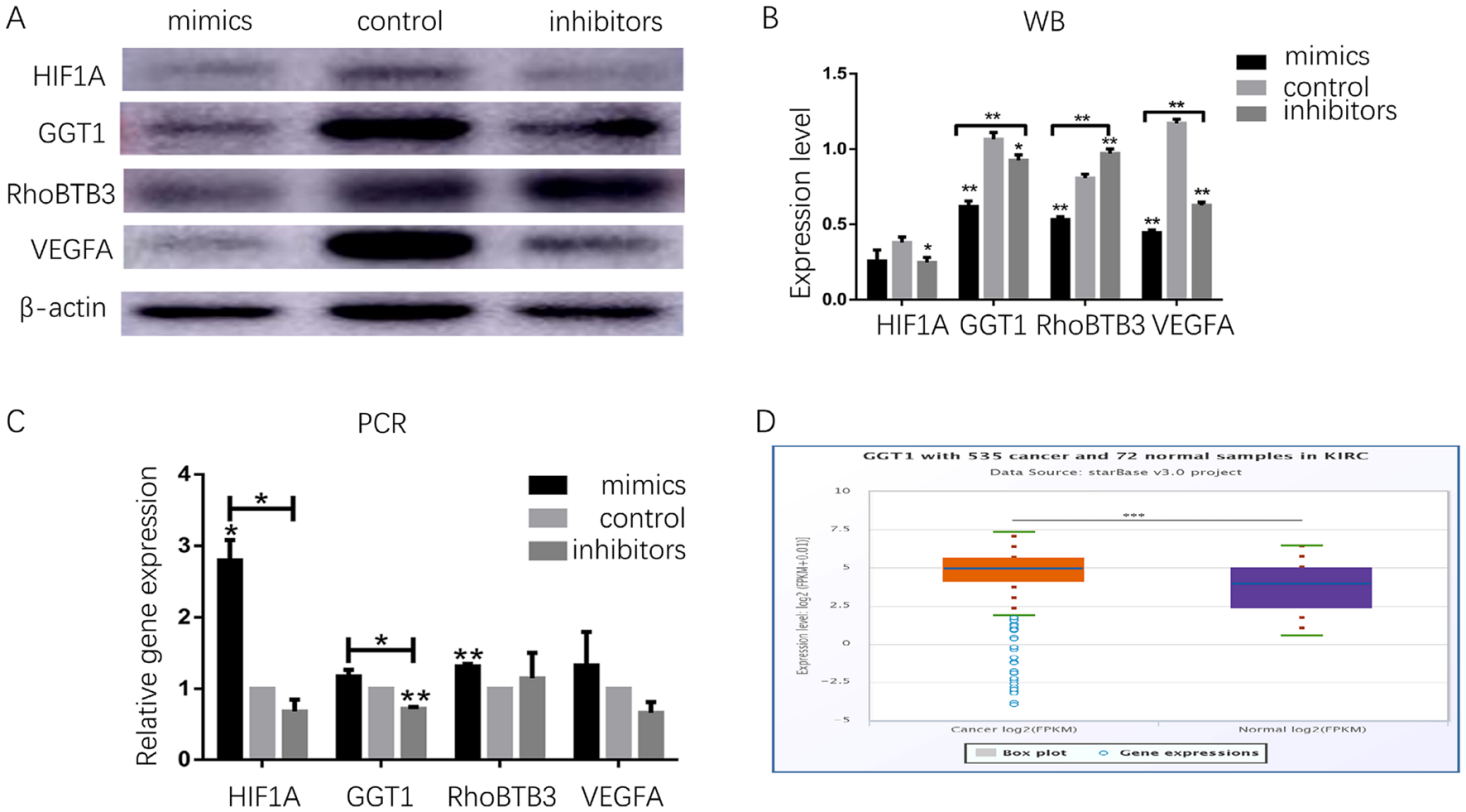
miR-142-3p regulates RCC migration and invasion via hypoxia and GSH pathways by targeting RhoBTB3. 786-O cells were transfected with miR-control, miR-142-3p-mimics, and, miR-142-3p-inhibitors. Protein expression of RhoBTB3, hypoxia, and GSH pathway-related biomarkers, including HIF1A, VEGFA, and GGT1 using Western blot (**A**) (the original blots/gels are presented in Supplementary Fig. 3, the samples derive from the same experiment and that gels/blots were processed in parallel). The mRNA and protein expression of RhoBTB3, HIF1A, VEGFA, and GGT1 in 786-O cells was determined using qRT-PCR and Western blot, respectively (**B**,**C**). Expression of GGT1 differed significantly between RCC and the normal adjacent tissues (enhanced in KIRC, **D**, *P* < 0.001).

Since the metastasis potential is critical for RCC progression, we explored whether miR-142-3p regulates the migration and invasion in 786-O cells. Next, we investigated whether RhoBTB3 is involved in this process; the levels of RhoBTB3 were determined using qRT-PCR and Western blot. Strikingly, the miR-142-3p-inhibitors significantly increased the expression of RhoBTB3, whereas the miR-142-3p-mimics remarkably suppressed RhoBTB3 protein levels in 786-O cells (Fig. 4A,B; *P* < 0.01). Strikingly, the expression of mRNA levels was not correlated with protein levels.

Furthermore, we evaluated the critical role of hypoxia pathways in miR-142-3p-mediated metastasis and invasion in RCC. Western blot and qRT-PCR (Fig. 4C; *P* < 0.05) results showed that the miR-142-3p-inhibitors decreased the expression of HIF1A and VEGFA. Conversely, the miR-142-3p-mimics increased the mRNA expression of *HIF1A* and *VEGFA*, as assessed by qRT-PCR (Fig. 4C).

Interestingly, the mRNA expression of *GGT1* was positively correlated with miR-142-3p (Fig. 4C; *P* < 0.05), and the miR-142-3p-inhibitors decreased the protein level of GGT1 (Fig. 4B; *P* < 0.05). Next, we determined the expression level of GGT1 in all subtypes of RCC by using starbase. As shown in Fig. 4D and Supplementary Fig. 4A, higher GGT1 expression was detected in KIRC (*P* < 0.001) and KIRP (*P* < 0.01) samples compared to the adjacent normal tissues, while significantly lower GGT1 expression was detected in KICH (*P* < 0.001; Supplementary Fig. 4B).

### Bioinformatics analysis the correlated pathways of miR-142-3p

**Figure 5:**
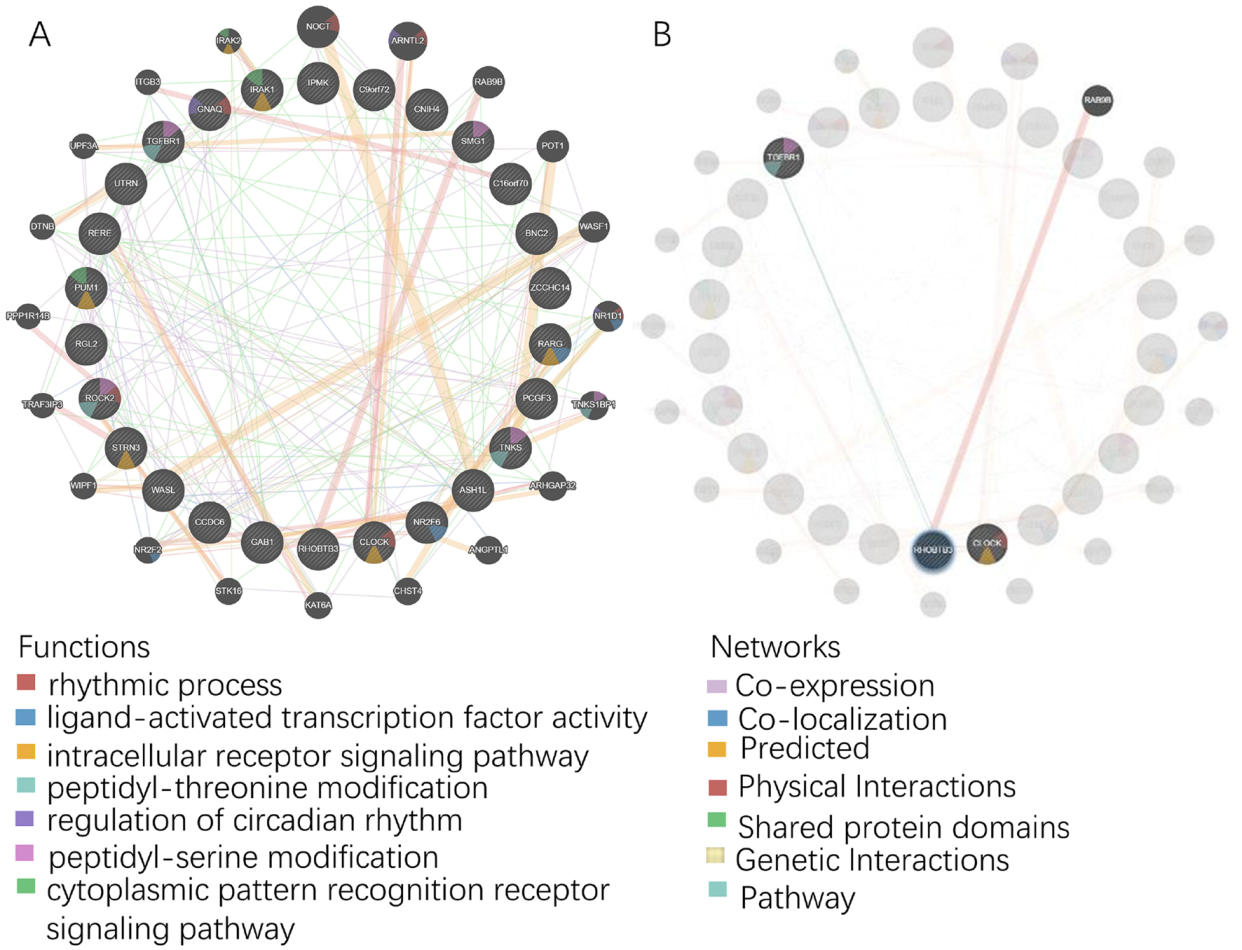
GENEMIA. (**A**) Predicted network of enriched terms across 26 genes which were predicted as miR-142-3p targeted genes in all three databases; (**B**) Physical interactions of RhoBTB3 and shared protein domains with Rab9b; co-localization and genetic interactions with TGFβR1; co-expression with CLOCK. The codes can be verified from the GENEMIA database.

Next, we performed a gene ontology (GO) analysis to assess whether microRNAs targeting multiple mRNAs from one pathway regulate cellular phenotype^33^. As shown in Fig. 5A, GENEMIA database showed that some pathways were related to miR-142-3p: circadian rhythm, rhythmic process, ligand-activated transcription factor activity, intracellular receptor signaling pathway, peptidyl-threonine modification, telomere organization, telomere maintenance, regulation of circadian rhythm, peptidyl-serine modification, and cytoplasmic pattern recognition receptor signaling pathway. Subsequently, as shown in Fig. 5B, we found a physical interaction between RhoBTB3 and Rab9b, co-localization and genetic interaction with TGFβR1, and co-expression with CLOCK. Moreover, the RhoBTB3-TGFβR1 pathways were associated with ligand-activated transcription factor activity and peptidyl-serine modification, while those of CLOCK were linked to rhythmic process and intracellular receptor signaling pathway. A previous study showed that RhoBTB3 was involved in retrograde transport from endosomes to Golgi apparatus^34^. These phenomena might partially explain our results that miR-142-3p targeting RhoBTB3 is involved in tumorigenesis and intracellular material transport. It could also be deduced that miR-142-3p targeting RhoBTB3 bound to TGFβR1 participating in ligand-activated transcription factor (for instance, TGFβ) pathways underlying tumor progression or with CLOCK participating in tumor radiochemotherapy resistance (especially in KIRC).

### Hypothesis

**Figure 6:**
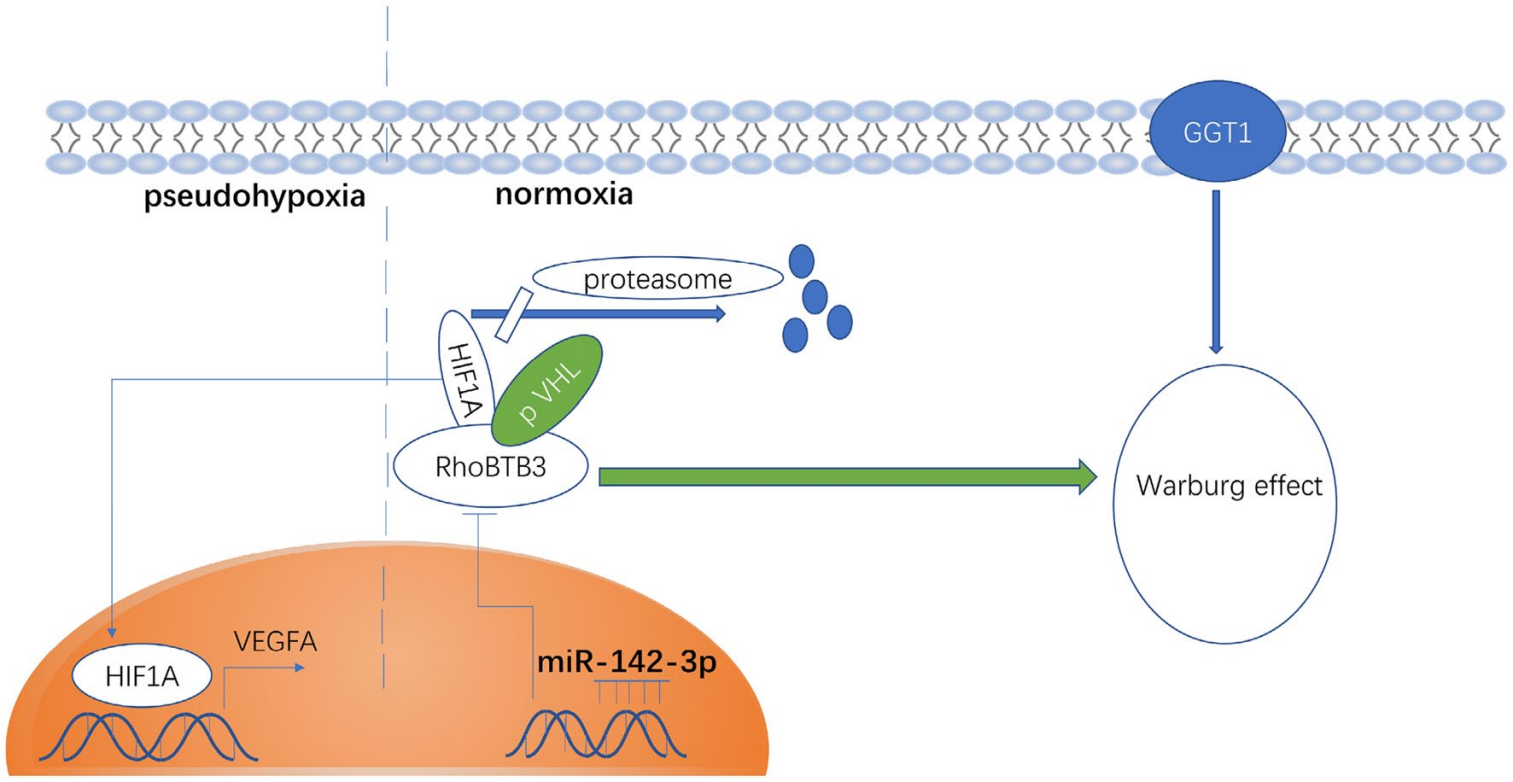
The hypothesis of the mechanism of microRNA-142-3p promotes renal cell carcinoma progression.

As shown in Fig. 6, a simplified hypothesis model depicts that miR-142-3p targets RhoBTB3 to downregulate its expression in the cytoplasm and then suppress the ubiquitination and degradation of HIF1A. The accumulation of HIF1A causes pseudohypoxia and the Warburg effect. In pseudohypoxia, HIF1A enters into the nucleus to enhance the expression of VEGFA. The Warburg effect maintains the cellular redox homeostasis and low reactive oxygen species (ROS) formation through GGT reaction with cytoplasm GSH to enhance tumor growth and chemoresistance. RhoBTB3 may crosstalk with the TGFβ pathway via TGFβR1 and promote epithelial-mesenchymal transition (EMT) to enhance cell growth and metastasis. However, the underlying mechanisms need to be investigated further.

## Discussions

RCC accounts for 3% of all adult malignancies and is the 13th most common malignancy diagnosed worldwide annually^35^. KIRC is the most common histological subtype of RCC. Hitherto, there is no biomarker in clinical application to predict the prognosis of RCC patients. Due to the significant roles of miRNAs and the targeting genes in the diagnosis and prognosis of cancer, increasing efforts are dedicated to the development of miRNA-based therapies. Using bioinformatics tools, we discovered that miR-142-3p was significantly elevated in KIRC and KIRP tissues compared to the adjacent normal tissues. Moreover, the level of miR-142-3p was elevated in total renal tumor compared to the normal tissues, and this high level was associated with the poor survival of patients with KIRC.

miRNAs regulate gene expression by binding to the 3′-untranslated region (UTR) of the target genes. In the current study, the target gene of miR-142-3p was identified using the bioinformatics analysis tools, and RhoBTB3 was screened as the candidate target as it was the highest hit by miRDB database. It is a novel gene, and this is the first study on RhoBTB3 and miRNA regulation. According to the in silico target prediction, the expression of RhoBTB3 was significantly reduced in KIRC, KIRP, and KICH. Furthermore, the negative correlation between miR-142-3p and RhoBTB3 in KIRC suggested that miR-142-3p and RhoBTB3 had an inverse function in RCC. Our results confirmed this conclusion. Firstly, the dual luciferase reporter assay demonstrated the direct interaction between RhoBTB3 and miR-142-3p. Furthermore, the mice xenograft model revealed that the inhibition of miR-142-3p restrained tumor metastasis, whereas the miR-142-3p-mimics promoted tumor metastasis. In addition, the miR-142-3p-mimics suppresses RhoBTB3 and vice versa. In vitro, the current data showed that inhibition of miR-142-3p promoted the protein level of RhoBTB3, while the miR-142-3p-mimics suppressed the protein level of RhoBTB3. Herein, we identified that miR-142-3p functions as an oncogene by targeting RhoBTB3 in the progression of RCC.

Strikingly, the mRNA level is not correlated with the protein level under miR-142-3p interference, suggesting that miR-142-3p regulates the translation of RhoBTB3. The classical miRNA function is synonymous with the posttranscriptional repression of target protein genes. The critical function of miRNAs in gene expression modulation is highlighted by the point that an individual gene is concurrently regulated by several miRNAs, and each miRNA can modulate the expression of several targets that have sequence complementarity with its seed region^36^. Several studies have reported the functions of miRNAs outside this paradigm, and some of these novel modes of regulation of gene expression have been implicated in cancers^37^, indicating that differential expression, maturation, or the stability of the host gene and the miRNA might vary. miRNAs inhibit as well as upregulate the transcription in a cell cycle-dependent manner^38^. The gene knockout and rescue tests of miR-142-3p and RhoBTB3 should be done to further confirm that RhoBTB3 is the direct target gene of miR-142-3p in RCC. Since miRNA inhibitors and mimics have different chemistry, future study should be done with the different kind of oligos suitable for each be used as the control group to elucidate the underlying mechanism.

Notably, the gene pathways involved in RCC tumorigenesis include the Von Hippel–Lindau (VHL)/hypoxia-inducible factor (HIF) and vascular endothelial growth factor (VEGF) pathways. The defects in the *VHL* gene are the most common cause of familial KIRC, and > 80% of patients with sporadic KIRC have an inactive gene^39^. In the case of hypoxia or loss of VHL function, HIF is stabilized, which leads to the characteristic hypoxia response, including activation of genes involved in angiogenesis, invasion, metastasis, cancer stemness, and metabolic reprogramming^40,41^. Interestingly, our data showed that inhibition of miR-142-3p suppressed the mRNA and protein levels of HIF1A and VEGFA in 786-O cells. Conversely, the miR-142-3p-mimics promotes the mRNA levels of *HIF1A* and *VEGFA*. In vivo, IHC showed that the expression of VEGFA was positively correlated with miR-142-3p interference. These data indicated that miR-142-3p promotes RCC by modulating HIF-1A/VEGFA axis.

In addition to HIF regulation, pVHL negatively modulates the TGFβ pathways in KIRC^42^. Since VHL loss is intimately associated with the genesis of KIRC, basal TGFβ activity is detected in KIRC tumors. TGF-βR1 plays a major role in functional crosstalk between TGFβ signaling pathway and hypoxia^42^. The inhibition of TGFβR1 does not affect cell proliferation but reduces the invasive capacity of KIRC cells^43^. The current data from GENEMIA database predicted the genetic interaction between RhoBTB3 and TGFBR1. RhoBTB3 may act as an intermediate in the crosstalk between the two signaling pathways. However, the underlying mechanisms need to be further investigated.

Genetic instability, mutagenesis, aberrant gene expression, and altered signaling pathways cause a glycolytic switch in 70–80% of human cancers leading to aerobic glycolysis (the Warburg effect). The molecular and functional processes of the Warburg effect include maintenance of the cellular redox homeostasis and low ROS formation. The glycolytic switch is an early event in oncogenesis and primarily supports cell survival. Delineating tumor metabolism for specific cancers is crucial to establish their unique signatures of biosynthetic and energy demands. Moreover, understanding metabolic reprogramming also provides functional imaging opportunities based on the altered pathways^44^. Most forms of kidney cancer show changes in oxygen sensing and glutamine^45,46^. GSH pathway is essential for KIRC progression. A study highlighted the role of GGT1 and GSH pathways in regulating proliferation, migration, and therapeutic sensitivity of KIRC cells^18^. Interestingly, KICH, accounting for 5% of all renal tumors, exhibits significantly lower GGT1 levels than normal kidneys. Nevertheless, GGT1 inhibition also enhances KICH cell sensitivity to oxidative stress in other kidney cells^47^. Consistent with our findings, the expression of GGT1 differed significantly in RCC compared to adjacent normal tissues. Additionally, high GGT1 levels are correlated with poor patient survival in those suffering from renal, prostate, and ovarian cancers^14,15,48^. Our previous study results^17^ are consistent with these. The upregulated GGT expression in cancer protects the cancer cells against oxidative stress by increasing the intracellular GSH level, thereby supporting their growth and survival^49^. Also, the metabolism of GSH can exert pro-oxidant effects via GGT^50^. The upregulation of GGT might impose an increased oxidative burden on the cell, resulting in GSH consumption and a decrease in the cellular GSH reserves. The continuous production of ROS caused by increased GGT expression might contribute to genetic instability and tumor progression^48^. GGT1/GSH pathway inhibition enhances the efficacy of standard chemotherapeutic agents. For example, ovarian cancer cells overexpressing GGT1 are resistant to chemotherapies, such as cisplatin^51^ and 5-fluorouracil^52^, indicating that the development of a potent inhibitor of GGT1 might represent a new therapeutic strategy. Our data showed that miR-142-3p inhibition suppressed the mRNA and protein levels of GGT1 in 786-O cells. Conversely, the miR-142-3p-mimics could promote the mRNA levels of GGT1. These phenomena indicated that miR-142-3p promotes RCC by modulating the GGT1/GSH pathways. However, the underlying mechanisms need to be investigated further.

EVs are micro-vesicles, 40–150 nm diameter, secreted from various cells^53^. Several proteins, miRNAs, RNAs, and DNAs are contained in the exosomes, and their molecular signature reflects their cell origin. The exosomes exist in body fluids, such as blood and urine, and are expected as novel markers for various diseases, including cancer. The search of the BB cancer database revealed that the miR-142-3p level in EVs was significantly reduced in renal tumors compared to the normal tissues. In our previous study^17^, we found that preoperative elevation of serum GGT is associated with poor prognosis in almost all pathologic types of RCC, including lymph node metastasis and/or distant metastasis. The serum and urine miRNAs act as novel biomarkers can be implemented due to their non-invasiveness and wide application, prompting a new direction for future research.

## Conclusions

Taken together, these data indicated that miRNA-142-3p promotes RCC progression by targeting RhoBTB3 to regulate HIF-1 signaling and GGT/GSH pathways. However, further investigation is needed to test the value of these genes, provide a novel insight into renal cancer progression and chemoresistance, and identify the critical therapeutic targets.

## Supplementary Information

Supplementary Figures.

## Data Availability

The datasets generated for this study are available on request to the corresponding author. The review of miR-142-3p, RhoBTB3, and GGT1 expression in RCC are available in the StarBase [ENCORI: The Encyclopedia of RNA Interactomes. (sysu.edu.cn)]. The correlations between miR-142-3p and gene expression data of predicted targets are available in TargetScan (TargetscanHuman 8.0: predicted miRNA targets of miR-142-3p.1), DIANA [DIANA tools—Tarbase v8 (uth.gr)], and miRDB (miRDB Search Result). The miR-142-3p’s mature sequences targeting of human RHOBTB3 are available in the TargetScan (TargetScanHuman 8.0 predicted targeting of Human RHOBTB3). Furthermore, the target genes predicted by all three databases with the target score 70–100 were identified using the venn diagram which are available in the genevenn [GeneVenn (sourceforge.net)]. The functions involving the predicted genes are available in GENEMANIA (GeneMANIA). The expression levels of exosome and tissue miR-142-3p are available in BBCancer [BBCancer (renlab.org)].

## Abbreviations

RCC: Renal cell carcinoma
KIRC: Kidney renal clear cell carcinoma
KIRP: Kidney renal papillary cell carcinoma
KICH: Kidney chromophobe
(p)VHL: Von Hippel–Lindau gene/protein
GGT: Gamma-glutamyl transpeptadase
GSH: l-Glutathione
VEGFA: Vascular endothelial growth factor A
HIF1A: Hypoxia-inducible factor 1-alpha
TGFβ: Transforming growth factor beta
TGFβR1: Transforming growth factor beta receptor 1
