# Supplementary material for: MicroRNA-142-3p promotes renal cell carcinoma progression by targeting RhoBTB3 to regulate HIF-1 signaling and GGT/GSH pathways
Source: Sci Rep. 2023 Apr 12;13:5935. doi: 10.1038/s41598-022-21447-2 (PMC10097650; doi:10.1038/s41598-022-21447-2)
Supplement: Supplementary file 1 — Supplementary Figures. [file 41598_2022_21447_MOESM1_ESM.pdf]

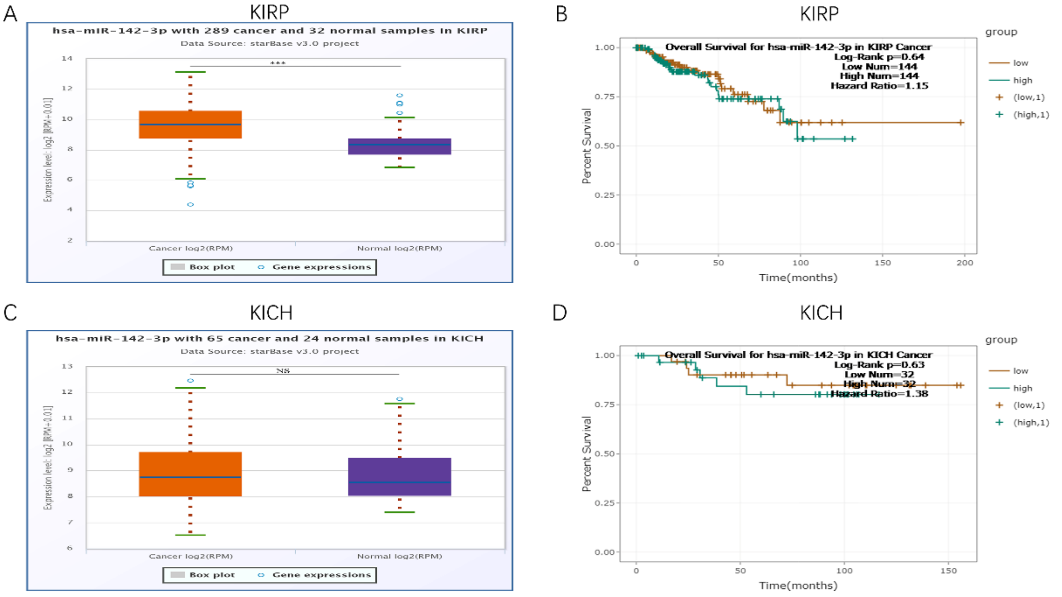

Supplementary Figure 1. Expression pattern of miR-142-3p in clinical samples. Expression of miR-142-3p in KIRP (A, $P<0.001$), and KICH (C, $P>0.05$) samples compared to adjacent normal tissues. Kaplan–Meier survival curves analysis for patients in TCGA KIRP (B, $P<0.01$), and KICH (D, $P>0.05$) cohort (Orange=low, Blue=high).

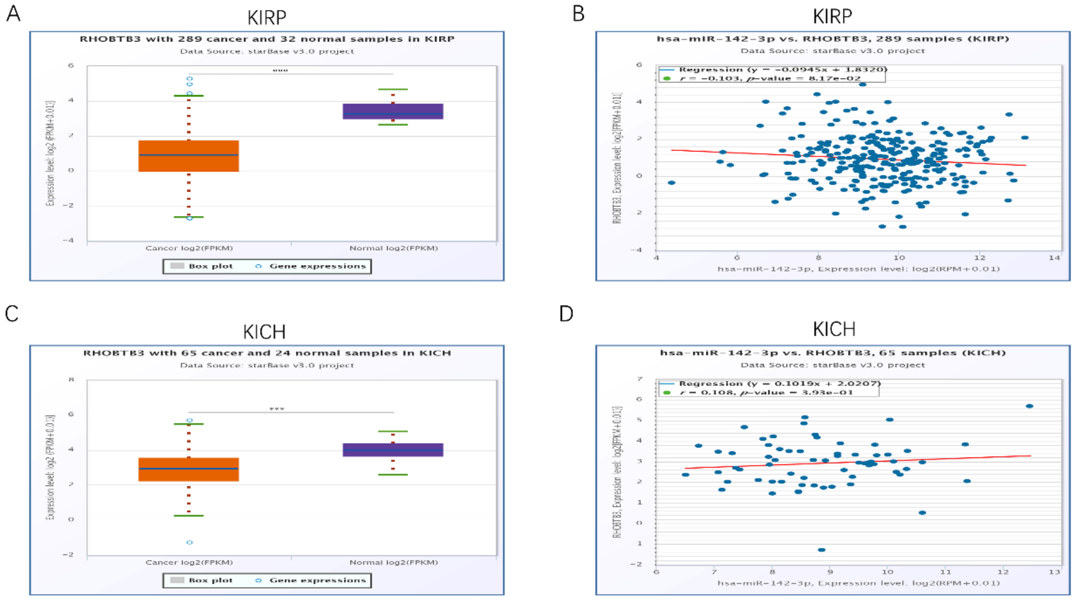

Supplementary Figure 2. Database analysis of miR-142-3p and RhoBTB3
The expression of RhoBTB3 was reduced significantly in KIRP (A, $P<0.001$), KICH (C, $P<0.001$). In KIRP (B, $P>0.05$) and KICH (D, $P>0.05$), the correlation between the miR-142-3p and RhoBTB3 did not show a significant difference.

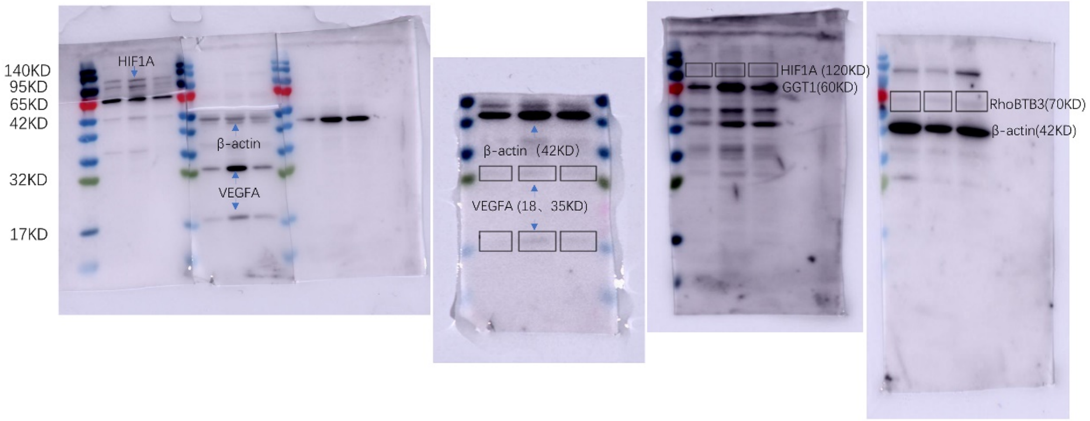

Supplementary Figure 3. miR-142-3p regulates RCC migration and invasion via hypoxia and GSH pathways by targeting RhoBTB3

786-O cells were transfected with miR-142-3p-control, -mimics, and -inhibitors. Protein expression of RhoBTB3, hypoxia, and GSH pathway-related biomarkers, including HIF1A, VEGFA, and GGT1 using Western blot.

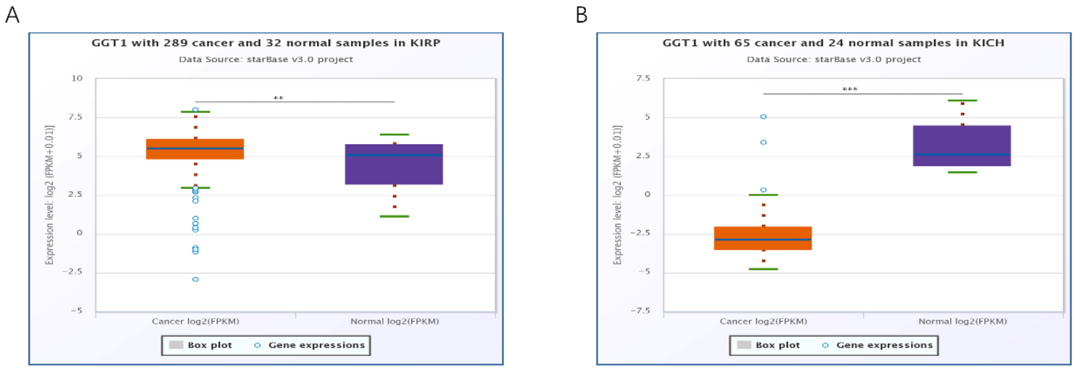

Supplementary Figure 4. miR-142-3p regulates RCC migration and invasion via GSH pathways by targeting RhoBTB3

Expression of GGT1 differed significantly between RCC and the normal adjacent tissues (enhanced in KIRP, A, $P<0.01$; reduced in KICH, B, $P<0.001$).
